# Necrotizing enterocolitis is associated with acute brain responses in preterm pigs

**DOI:** 10.1186/s12974-018-1201-x

**Published:** 2018-06-09

**Authors:** Jing Sun, Xiaoyu Pan, Line I. Christiansen, Xiao-Long Yuan, Kerstin Skovgaard, Dereck E. W. Chatterton, Sanne S. Kaalund, Fei Gao, Per T. Sangild, Stanislava Pankratova

**Affiliations:** 1 Comparative Pediatrics and Nutrition, Department of Veterinary and Animal Sciences, University of Copenhagen, DK-1870 Frederiksberg C, Denmark; 2 Department of Biotechnology and Biomedicine, Technical University of Denmark, DK-2800 Kgs. Lyngby, Denmark; 3 Department of Food Science, University of Copenhagen, DK-1958 Frederiksberg C, Denmark; 4 Research Laboratory for Stereology and Neuroscience, Bispebjerg-Frederiksberg Hospitals, DK-2400 Copenhagen, Denmark; 5 Agricultural Genomics Institute at Shenzhen, Chinese Academy of Agricultural Sciences, 518000, Shenzhen, China; 6 Department of Pediatrics and Adolescent Medicine, Rigshospitalet, Blegdamsvej 9, DK-2100 Copenhagen, Denmark; 7 Laboratory of Neural Plasticity, Department of Neuroscience, University of Copenhagen, DK-2200 Copenhagen, Denmark

**Keywords:** Preterm birth, Necrotizing enterocolitis, Hippocampus, Neurite outgrowth, Neuroinflammation, S100A9, Cerebrospinal fluid, Motor activity

## Abstract

**Background:**

Necrotizing enterocolitis (NEC) is an acute gut inflammatory disorder that occurs in preterm infants in the first weeks after birth. Infants surviving NEC often show impaired neurodevelopment. The mechanisms linking NEC lesions with later neurodevelopment are poorly understood but may include proinflammatory signaling in the immature brain. Using preterm pigs as a model for preterm infants, we hypothesized that severe intestinal NEC lesions are associated with acute effects on the developing hippocampus.

**Methods:**

Cesarean-delivered preterm pigs (*n* = 117) were reared for 8 days and spontaneously developed variable severity of NEC lesions. Neonatal arousal, physical activity, and in vitro neuritogenic effects of cerebrospinal fluid (CSF) were investigated in pigs showing NEC lesions in the colon (Co-NEC) or in the small intestine (Si-NEC). Hippocampal transcriptome analysis and qPCR were used to assess gene expressions and their relation to biological processes, including neuroinflammation, and neural plasticity. Microglia activation was quantified by stereology. The neuritogenic response to selected proteins was investigated in primary cultures of hippocampal neurons.

**Results:**

NEC development rapidly reduced the physical activity of pigs, especially when lesions occurred in the small intestine. Si-NEC and Co-NEC were associated with 27 and 12 hippocampal differentially expressed genes (DEGs), respectively. These included genes related to neuroinflammation (i.e., *S100A8*, *S100A9*, *IL8*, *IL6*, *MMP8*, *SAA*, *TAGLN2*) and hypoxia (i.e., *PDK4*, *IER3*, *TXNIP*, *AGER*), and they were all upregulated in Si-NEC pigs. Genes related to protection against oxidative stress (*HBB*, *ALAS2*) and oligodendrocytes (*OPALIN*) were downregulated in Si-NEC pigs. CSF collected from NEC pigs promoted neurite outgrowth in vitro, and the S100A9 and S100A8/S100A9 proteins may mediate the neuritogenic effects of NEC-related CSF on hippocampal neurons. NEC lesions did not affect total microglial cell number but markedly increased the proportion of Iba1-positive amoeboid microglial cells.

**Conclusions:**

NEC lesions, especially when present in the small intestine, are associated with changes to hippocampal gene expression that potentially mediate neuroinflammation and disturbed neural circuit formation via enhanced neuronal differentiation. Early brain-protective interventions may be critical for preterm infants affected by intestinal NEC lesions to reduce their later neurological dysfunctions.

**Electronic supplementary material:**

The online version of this article (10.1186/s12974-018-1201-x) contains supplementary material, which is available to authorized users.

## Background

Necrotizing enterocolitis (NEC) is an acute devastating intestinal inflammatory disease that mainly occurs in preterm infants shortly after birth [1]. Epidemiological studies show that 45% of NEC survivors were neurologically impaired at 20 months of age with a higher risk of cerebral palsy, hearing, visual, cognitive, and psychomotor impairments [2, 3]. Severe NEC requiring surgery is an independent risk factor for severe brain injury detected on MRI, poor mental and psychomotor development at around 2 years of age [4, 5], and various cognitive deficits at school age [6]. It is conceivable that NEC may cause acute damage in the developing brain and subsequent lasting neurodevelopmental disorders. Yet, the underlying mechanisms and NEC-associated acute brain effects are largely unknown.

NEC in preterm infants is characterized by excessive gut inflammation, potential pathogen leakage, and systemic inflammation [1]. Neonatal inflammation may adversely affect the processes of maturation of neuronal and immune systems in a critical period of development [7, 8]. Studies have shown that early life exposure to lipopolysaccharide (LPS) leads to reduced hippocampal volume, dysregulated neurogenesis, increased number of microglia cells, axonal injury, and memory impairment in rats [9–11]. Similarly, chronic intestinal inflammation reduces hippocampal neurogenesis in mice [12] and neonatal viral infection induces hippocampal neuroinflammation and learning impairments in piglets [13]. This may be mediated by inflammatory cytokines which are normally expressed in the developing brain and regulate processes of neurogenesis, neuronal migration, synaptogenesis, and synaptic plasticity [14–16]. A dual role of proinflammatory cytokines in the developing brain may relate to activation of the ubiquitous NFκB signaling pathway, which in neurons is responsible for plasticity and survival, whereas in glial cells, it is involved in mediation of pro-inflammatory responses [17]. Such neurodevelopmental and neurodegenerative abnormalities may also induce changes in the composition of cerebrospinal fluid (CSF) [16, 18, 19], but few studies have investigated the links among brain damage, CSF, and NEC in preterm neonates [20].

Preterm pigs delivered at 90% gestation show impaired gut, immune, and brain development with a high sensitivity to neonatal infections (i.e., NEC and sepsis) and behavioral and learning deficits [21–23]. Thus, a large proportion of preterm pigs develop NEC spontaneously within the first week after birth when fed sub-optimal diets (e.g., infant formula or human donor milk) [24]. In contrast to rodent models, this model does not require excessive hypothermia and hypoxia treatments [21]. Using preterm pigs as a model for preterm infants, we hypothesized that NEC lesions would induce immediate changes to the developing hippocampus that might help to explain the later neurodevelopmental deficits in NEC survivors. We show that specifically NEC lesions located to the small intestine are associated with reduced physical activity and upregulation of inflammation-related genes in the hippocampus. Exposure of hippocampal neurons to CSF from pigs with NEC promoted neurite outgrowth in vitro, maybe via NEC-related factors in CSF, such as VEGF, CINC-3, and S100A9 proteins. Thus, our results support the hypothesis that NEC lesions lead to immediate effects on the developing brain in preterm infants.

## Methods

### Spontaneous NEC model in preterm pigs

One hundred and seventeen preterm piglets were delivered from eight sows by cesarean section at day 106 (90% of gestation, Danish landrace x Large White x Duroc, Askelygaard farm, Denmark). Pigs were housed in individual incubators with regulated temperature (37–38 °C) and oxygen supply (0.5–2.1/min, within the first 24 h). Pigs were inserted with an orogastric feeding tube and an umbilical catheter for parental nutrition and sow plasma infusion, as described previously [25]. To induce spontaneous NEC [21], preterm pigs were fed with gradually increasing doses of different types of human donor milk (0–135 mL/kg/day) and gradually decreasing doses of parenteral nutrition (96–48 mL/kg/day) for 8 days, as described previously [24, 25].

### Home cage activity and neonatal arousal recordings

During the study period, the physical activity for all piglets was recorded using infrared video cameras connected to a motion detection recorder. The proportion of active time was analyzed with PIGLWin application software (Ellegaard System, Faaborg, Denmark), as described previously [26]. The neonatal arousal of each piglet was registered as the time from birth to the first opening of eyes, first standing, and first walking, as described previously [26].

### Tissue collection, NEC evaluation, and gut cytokine expression

Pigs were anesthetized, and blood was drawn by cardiac puncture, followed by euthanasia by an intracardiac injection of sodium pentobarbital (60 mg/kg). Heparinized plasma fractions were collected and stored at − 80 °C. The CSF samples were collected by sub-occipital puncture immediately after euthanasia, aliquoted, and stored at − 80 °C. Following determination of brain wet weight, the brain was quickly dissected and the left hippocampal formations were snap-frozen in liquid nitrogen and stored at − 80 °C until further processing. The right hemisphere was fixed in 4% paraformaldehyde. Brain dry weight and water content were determined after drying the remaining brain tissues to a constant weight.

The whole gastrointestinal tract (GIT) was excised, and the pathological lesions in the proximal, middle, and distal small intestine, and in the colon, were scored macroscopically by two independent investigators, using an established NEC severity scoring system: score 1: absence of macroscopic lesions; score 2: local hyperemia; score 3: hyperemia, mild hemorrhage, extensive edema; score 4: extensive hemorrhage; score 5: local necrosis and pneumatosis intestinalis; score 6: extensive transmural necrosis and pneumatosis intestinalis (Additional file 1: Figure S1 [24]). Pigs with score 1 in all regions were diagnosed as without having NEC (No NEC, *n* = 51). Pigs with a severity score ≥ 4 in the small intestine (with or without colon lesions) were diagnosed as severe small intestinal NEC (Si-NEC, *n* = 13). Pigs with a score ≥ 4 only in the colon were diagnosed as severe colon NEC (Co-NEC, *n* = 34). Detailed disease characteristics, group information, and number of animals included in further analyses are shown in Additional file 2: Table S1 and in the corresponding figure legends. Nineteen pigs scored with 3 in minimum one region across the small intestine and the colon showed borderline pathological lesions that may or may not reflect initial NEC lesions. To minimize the effects of such diagnostic uncertainty (which is common also for infants undergoing surgery for NEC), we excluded all these animals from further analyses, resulting in groups of pigs only with clear diagnosis as NEC or no NEC by macroscopic inspection. In preterm pigs, mild clinical symptoms related to NEC are most often located in the colon region while more severe NEC symptoms usually also involve the small intestine (with or without lesions in the colon) [26–28]. Expression of proinflammatory cytokines including IL-1β, IL-6, and IL-8 in the distal intestine and the colon were measured with porcine DuoSet ELISA kits (R&D Systems, Minneapolis, MN, USA), according to the manufacturer’s protocol and presented per milligram of total protein (Pierce BCA Protein Assay Kit, Thermo Fisher Scientific, Rockford, IL, USA).

### Plasma and CSF sample analysis

Spectrophotometric measurement of the oxyhemoglobin content of the CSF samples was performed at A450 nm to exclude samples containing blood contamination [29]. The total protein concentration in the CSF and plasma samples was measured with the Pierce BCA Protein Assay Kit. Plasma and CSF albumin, lactate, and glucose were measured with a GEM premier 3000 whole blood analyzer (Instrumentation Laboratory, Bedford, MA). Plasma C-reactive protein (CRP) concentration was measured by ELISA (DY2648, R&D systems, Minneapolis, MN, USA).

### Neurite outgrowth assay

Hippocampal neurons were isolated from Wistar rats (E18; Charles River, Sulzfeld, Germany), plated on eight-well Permanox Lab-Tek chamber slides (Nunc, Roskilde, Denmark) at a density of 10,000 cells/well as previously described [30] and immediately stimulated with CSF samples. Optimization experiments with serially diluted CSF samples showed that a final concentration of CSF in the culture medium of 1% was the most optimal, which was in accordance with previous work [31]. For each slide, unstimulated cells and cells treated with 3 μg/ml of the neurotrophic peptide Epobis were used as negative and positive controls, respectively [30]. In independent experiments, hippocampal neurons were stimulated with recombinant human VEGF, S100A8, S100A9, hetero-complex of S100A8/A9, and rrCINC-3 (CXCL2) (all from R&D Systems, Minneapolis, USA; rhS100A9 and rhS100A8 were kind gifts from Dr. J. Klingelhöfer, University of Copenhagen). Twenty-four hours after the stimulation, neurons were fixed in 4% *v*/*v* formaldehyde and stained with polyclonal rabbit growth-associated protein (GAP)-43 antibodies (1:1000; Millipore), followed by secondary Alexa Fluor 488- or 546-conjugated goat anti-rabbit antibodies (1:1000; Molecular Probes). Images were acquired using a Zeiss Axiovert 100 microscope connected with AxioCamMRm camera using the ZEN 2012 software. The quantification of neurite outgrowth and number of neurites per cell were performed as described previously [30].

### Hippocampal RNA-seq analyses

Intact frozen hippocampi (*n* = 5–6 per group) were homogenized by a cryogenic tissue pulverizer in liquid nitrogen, and total RNA was isolated with RNeasy Lipid Tissue Mini Kit (Qiagen, Copenhagen, Denmark). The integrity of RNA samples for RNA-seq and qPCR analyses was evaluated with Agilent Bioanalyzer 2100 and RNA 6000 Nano Chips (Agilent Technologies, Glostrup, Denmark) and resulted in an average RNA integrity number (RIN) of 8.5 (SD ± 0.6). Sequencing libraries were constructed using NEBNext UltraTM RNA library Prep Kit for Illumina (New England BioLabs, Ipswich, MA, USA), following the manufacturer’s recommendations. After amplification, products were purified with the AMPure XP system and library quality was assessed on the Agilent Bioanalyzer 2100 system. The clustering of the index-coded samples was performed on a cBot Cluster Generation System using a HiSeq 4000 PE Cluster Kit (Illumina, San Diego, CA, USA). After cluster generation, constructed cDNA libraries were sequenced on an Illumina Hiseq 4000 platform (Illumina) and 150-bp paired-end raw reads were generated.

Raw reads were trimmed to produce clean reads, including removal of the adapter sequence, and low-quality reads containing either more than 50% bases with *q* value < 10 or ≥ 10% N bases, as detected by the FASTX tool kit (v 0.0.13, http://hannonlab.cshl.edu/fastx_toolkit). Following this cleaning, 273,958,970 clean and paired reads were generated in total. All clean reads were aligned to the Sscrofa 10.2 genome and gene model annotation file (www.ensembl.org/Sus_scrofa/Info/Index) using Tophat (v2.1.1)-Cufflinks (v2.2.1) pipeline [32]. The expression level of each gene was estimated using Fragments Per Kilobases per Million reads (FPKM). According to Cuffdiff, the genes with a statistical *q* value < 0.2 were considered as differentially expressed genes (DEGs) [32]. Biological process enrichment was analyzed using the Cytoscape plug-in ClueGO [33], and enrichment tests with adjusted *p* values < 0.05 were considered significant.

### Validation of gene transcription by qPCR analyses

Expression of DEGs of interest and other related genes were further measured by microfluidic qPCR analyses. To ensure valid data, for each RNA sample, two separate technical cDNA replicates were synthesized and a non-reverse transcriptase control was included. Pre-amplification of each cDNA was carried out using TaqMan PreAmpMasterMix (Applied Biosystems, Foster City, CA, USA) followed by exonuclease treatment (Exonuclease 1, New England biolabs, PN MO293L), as described previously [34]. Porcine-specific primers were designed whenever possible over introns (Primer3: http://frodo.wi.mit.edu; Sigma-Aldrich, Broendby, Denmark). Gene symbol, primer sequences, and amplicon lengths are shown in Additional file 3: Table S6. The amplification efficiencies of all primers were between 85 and 115%. Quantitative PCR of pre-amplified cDNA samples, including non-reverse and non-template controls, was performed using 96.96 Dynamic Array Integrated Fluidic Circuits on a BioMark thermocycler (Fluidigm, CA, USA). The cycling conditions were 2 min at 50 °C, 30 min at 80 °C for thermal mix, then 2 min at 50 °C and 10 min at 95 °C, followed by 35 cycles of 15 s at 95 °C and 1 min at 60 °C for the signal detection. Melting curves were generated after each run (from 60 to 95 °C, increasing 1 °C/3 s). Acquired Cq values were uploaded to the online PCR analysis tool (http://dataanalysis.sabiosciences.com/pcr/arrayanalysis.php) and analyzed as previously described [35]. Using GenEx, the expression levels of target genes were normalized to the three reference genes including *GAPDH*, *RPL13A*, and *ACTB*.

### Stereology

Entire hippocampi were dissected from fixed hemispheres, embedded in paraffin using a Leica ASP300 S tissue processor, and sectioned with a Jung HN40 sliding microtome for 5-μm exhaustive sagittal sections. Sections were sampled using uniform random sampling [36], by which every 70th section pair was collected herein yielding 8–12 section pairs per hippocampus. For immunohistochemistry, the sampled sections were dewaxed and hydrated followed by incubation in 3% H_2_O_2_ to block endogenous peroxidase activity. Antigens were retrieved by boiling the sections for 15 min in 10 mmol/l citrate buffer (pH 6). Sections were stained with anti-Iba1 antibodies (1:1000; ab5076, Abcam, Denmark) followed by HRP-conjugated secondary antibody (1:500; Polyclonal Rabbit Anti-Goat/HRP, P0449, Dako, Denmark). The reaction was developed using 3,3′-Diaminobenzidine (Sigma-Aldrich, Denmark), then sections were counter-stained with Mayer’s hematoxylin, mounted with Pertex, and cover slipped (Leica Microsystems, Ballerup, Denmark).

The total number (*N*) of microglia was estimated using the physical disector-design [37] for three hippocampi and counting on series of single-sections. The numerical density (*N*_V_) was estimated by dividing the total number of particles (∑*Q*) by the volume in which they were counted, e.g., area of the frame (*a*(frame)), height of the section (*h*), and number of disectors (∑*P*) (Eq. 1: *N*_*V*_ *= ∑Q*/(*a*(frame)*h*∑P)*.* To estimate the total number (*N*) of microglia in the region, the numerical density (*N*_*V*_) was multiplied by the volume of the region of interest (ROI), i.e., the reference volume (*V*_ref_) (Eq. 2: *N=N*_V_*V_ref_). The *V*_ref_ was estimated by Cavalieri’s method, where the number of points hitting the ROI (∑*p*) was multiplied by the area per point (*a*(point)) and the block advance (BA) (Eq. 3: *V*_ref_ *= a*(point)*BA*∑p). As the estimated bias introduced by counting cells in only one of the sections in a section pair was 3.1%, the number of microglia (*N*) on a series of single sections was calculated as described above in Eqs. 1–3. From the systematic uniform random sampled images, the ratio of amoeboid to total microglia was estimated using following morphological criteria: cells with round or amoeboid shapes, with no processes, were classified as amoeboid microglia, which were distinguished from cells with a small or large cell body with visible thin or thick ramifications (ramified or primed microglia, respectively [38]). Cell counting was performed using the NewCast software (Visiopharm, Hoersholm, Denmark) and a Nikon Eclipse 60i microscope (Olympus, Ballerup, Denmark) equipped with a Heidenhain electronic microcator measuring the *z*-axis and a ProScan II motorized stage system (Prior Scientific Instruments., Cambridge, UK). Digital live microscope images were visualized by a high-resolution camera (Olympus DP72, Nikon Nordic AB, Copenhagen, Denmark).

### Statistics

Data analyses were performed using the software package R (version 3.3.2). Continuous outcomes (e.g., brain weight, pro-inflammatory cytokine levels) were analyzed using the *lm* function, and repeated measurements (i.e., physical activity) were analyzed using the *lmer* function. The normality and variance homogeneity of the residuals and fitted values were tested, and data transformation was performed if necessary. To determine any potential sex bias on the processes of brain development and neuroimmune responses, all above models were adjusted for potential covariates and confounders (i.e., birth weight, litter, and sex). Data was subsequently treated by Dunnett’s post hoc test with the No NEC group as the reference group using *glht* function. Neuritogenesis was analyzed by one-way ANOVA. Data were autoscaled and applied to principal components analysis (PCA) using the R package “pcaMethods” [39]. Data were presented as means ± SEMs, unless otherwise stated, and *p* < 0.05 was considered significant, with *p* < 0.15 indicated as a tendency to an effect.

## Results

### NEC is associated with reduced physical activity, gut and systemic inflammation

**Fig. 1:**
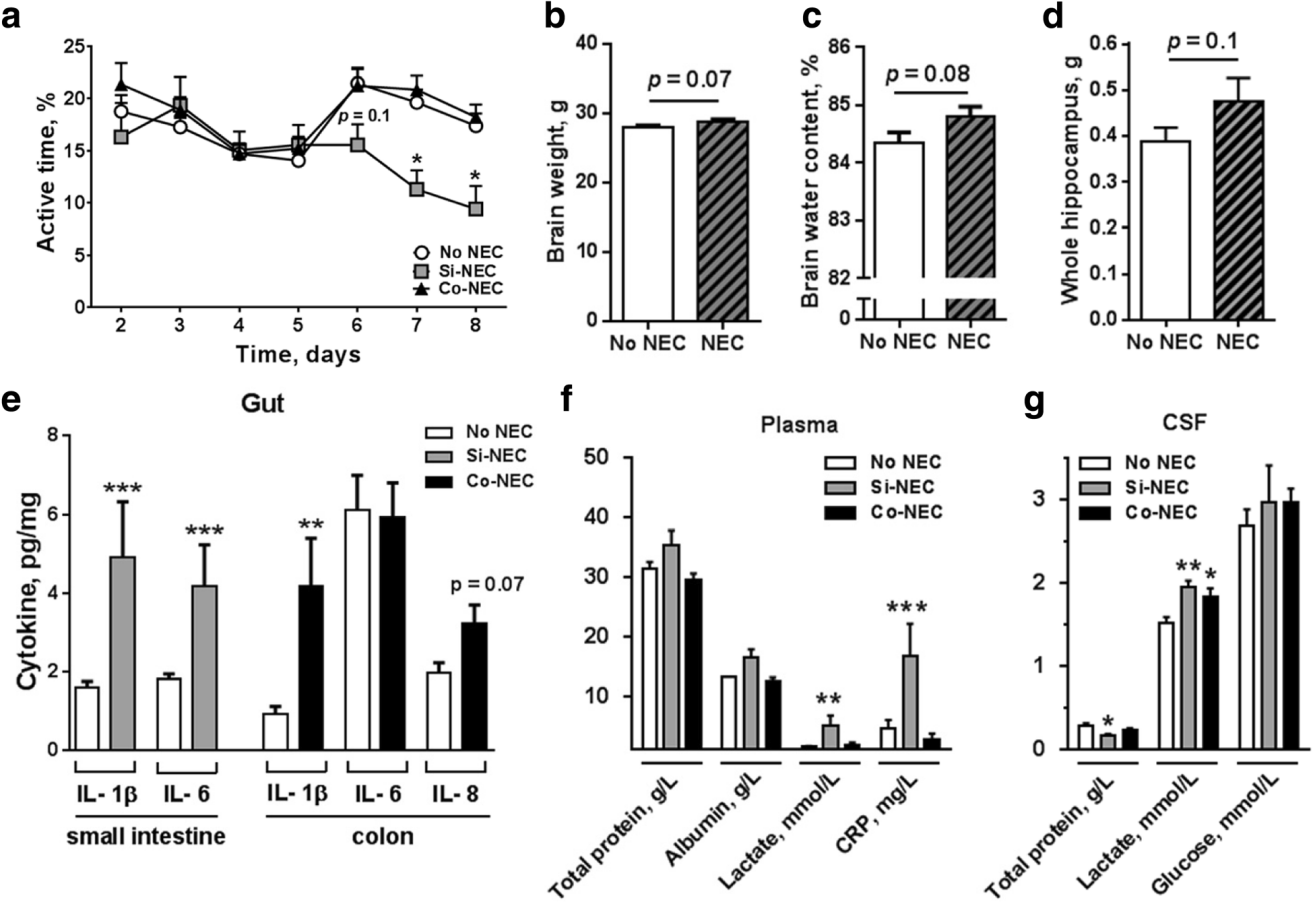
Gut cytokines, plasma and CSF biochemistry, brain parameters in NEC versus No NEC pigs. **a** Daily physical activity expressed as percentage of active time starting on postnatal day 2. **b** Brain weight, **c** water content, **d** hippocampal weight comparison, and **e** expression of proinflammatory cytokine IL-1β, IL-6, IL-8 in the distal small intestine and the colon. **f** Total protein, albumin, lactate, and CRP in the plasma. **g** Total protein, lactate, and glucose in the CSF. Values are means ± SEMs, *p < 0.05; **p < 0.01; ***p < 0.001, versus No NEC pigs

Firstly, we explored whether NEC was associated with altered physical activity. The time taken until the first stand, walk, or eyelid opening was similar between NEC and No NEC pigs (Additional file 2: Table S1). Specifically, the Si-NEC pigs showed a tendency to decreased physical activity beginning from day 6 (*p* = 0.1) and thereafter (*p* < 0.05), compared with No NEC pigs (Fig. 1a). Piglets with NEC in any region of the GIT tended to have higher brain wet weight (*p* = 0.07, Fig. 1b), probably due to higher hydration (*p* = 0.08, Fig. 1c). Among the compared brain regions (i.e., cerebrum, hippocampus, cerebellum, brain stem, and striatum), only the hippocampi tended to show increased wet weight in NEC-positive pigs (*p* = 0.1, Fig. 1d).

NEC diagnosis was supported by analyses of tissue pro-inflammatory cytokines [28], which revealed significant increases in IL-1β and IL-6 expression in the distal small intestine of Si-NEC animals (both *p* < 0.001), and elevations in IL-1β and IL-8 levels in the colon of Co-NEC animals (*p* < 0.01, *p* = 0.07, respectively, Fig. 1e). Considering the possible systemic effects of local gut inflammation, the concentrations of plasma CRP and lactate were higher in Si-NEC pigs than in No NEC pigs (*p* < 0.001, *p* < 0.01, respectively, Fig. 1f). Plasma glucose levels tended to be higher in Si-NEC pigs than in No NEC pigs (*p* = 0.06; Additional file 2: Table S1). Measurement of CSF metabolites indicated similar levels of glucose across the groups, whereas lactate levels were elevated in both the Si-NEC and Co-NEC groups (*p* < 0.05, Fig. 1g). Moderate increases of proinflammatory cytokines (i.e., VEGF, RAGE, MMP-8, IFN-γ, CINC-3, all above the 25% cutoff) were observed in the CSF from Si-NEC and Co-NEC pigs (Additional file 1: Figure S2).

### Cerebrospinal fluid from NEC pigs triggers neurite outgrowth in vitro

**Fig. 2:**
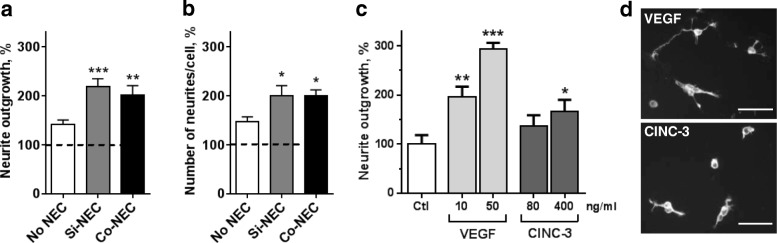
Neurotrophic effects of CSF from NEC versus No NEC pigs in primary hippocampal neurons. **a** Length of neurite and **b** number of primary neurites per cell were determined. Tested CSF samples were randomly selected from each group, *n* = 17/7/10 corresponding to No NEC/Si-NEC/Co-NEC. Values are means ± SEMs and were normalized to unstimulated controls (Ctl), which were set to 100% (dashed line; *p < 0.05; **p < 0.01; ***p < 0.001). **c** Primary neurons treated with recombinant VEGF (10 and 50 ng/ml) or CINC-3 (80 and 400 ng/ml). *p < 0.05; **p < 0.01; ***p < 0.001 versus Ctl. Data were generated from four to six independent experiments. **d** Representative micrographs of hippocampal neurons stained for GAP-43 to visualize growing neurites after stimulation with VEGF (50 ng/ml; top image) and CINC-3 (400 ng/ml; bottom image). Scale bar, 50 μm

CSF from healthy [40] and neurologically sick patients, including those with epilepsy [41], are known to promote viability and differentiation of neuronal cells. Moreover, a number of aforementioned NEC-associated CSF cytokines (Additional file 1: Figure S2) are known to promote neurite outgrowth, including INF-γ [42], MMP-8 [43], RAGE [44], and VEGF [45]. Therefore, to determine whether CSF factors in NEC-positive piglets might directly affect neuronal differentiation, we administered the CSF samples to primary hippocampal neurons, commonly used as a xenogeneic in vitro system to test the neurotrophic potential of biological fluids [40, 46]. CSF from No NEC pigs promoted neurite outgrowth to 141% of unstimulated controls (Fig. 2a; dashed line, set as 100%). Yet, compared with NEC-negative CSF samples, stimulation with CSF samples from either Si-NEC or Co-NEC pigs further promoted neuritogenesis to 200–220% (*p* < 0.05, Fig. 2a). Supporting these findings, treatment with CSF samples from both NEC groups of pigs resulted in a significantly higher number of neurites per cell, thus enhancing the sprouting of neurites (*p* < 0.05, Fig. 2b). To summarize, the neurite outgrowth effect of NEC-positive CSF samples strongly suggested that acute gut inflammation in preterm neonates may affect neuronal differentiation processes via specific CSF factors. We therefore tested two factors that were upregulated in CSF from both NEC groups, VEGF and CINC-3 (Additional file 1: Figure S2), for their ability to reproduce the neuritogenic effect of NEC-positive CSF samples in vitro. As shown in Fig. 2c, d, VEGF, and to a lesser extent, CINC-3, significantly promoted neurite outgrowth, compared with the control. Thus, VEGF and CINC-3 may be potential mediators of NEC-induced de-synchronization of neural circuit formation.

### Hippocampal transcriptome and biological processes that relate to NEC lesions

**Fig. 3:**
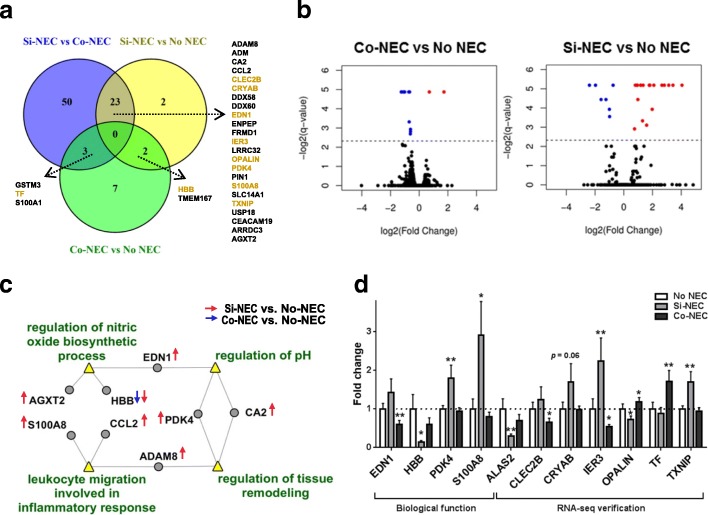
Hippocampal transcriptome in pigs with or without NEC. **a** Venn diagram indicates the number of hippocampal DEGs that were specifically related to Si-NEC (25 genes) and Co-NEC (10 genes), and two genes that were shared for both groups (*HBB* and *TMEM167*). These NEC-related DEGs are listed, including the genes further validated by qPCR in a larger cohort (highlighted in light brown, *n* = 9–16). **b** Volcano plots of RNA-seq hippocampal gene expression are shown as a scatter-plot of log_2_ changes in expression of NEC-affected pigs versus No NEC pigs plotted against the negative log of the *q* value (*n* = 5–6 per group). The genes up- or downregulated with FDR-adjusted statistical *q* value < 0.2 are shown as red and blue dots delineated by a dashed line. The transcriptional profiles for Si-NEC and Co-NEC pigs showed overlap in NEC-related effects for two genes, *HBB* and *TMEM 167B* (*p* < 0.0005). **c** Visualization of enriched biological processes defined by GO terms (yellow triangles), and Si-NEC and Co-NEC DEGs that are involved in the above biological processes are highlighted by red and blue arrows, correspondingly. **d** qPCR verification for selected NEC-related DEGs and DEGs were identified in biological functions at **c** by RNA-seq (*n* = 9–16 per group). Values are means ± SEMs. *p < 0.05; **p < 0.01 vs No NEC pigs (dashed line)

Neonatal infections are associated with long-term cognitive impairments [47]. Considering the role of the hippocampus in cognitive function and the rapid development of the hippocampus around birth, we suggested that early life insults, such as NEC, may be associated with acute hippocampal molecular changes. Thus, we performed a genome-wide profiling of hippocampal gene expression using RNA-seq. Among all the 23,293 genes detected in the pig genome, 13,904 genes passed quality control for DEGs testing according to Cuffdiff [32]. As a result, 0.64% of these genes (*n* = 89) were identified as DEGs in pairwise comparisons among the three groups, suggesting a marginal change in the genome-wide hippocampal gene expression that related to NEC diagnosis. Relative to No NEC, changes in the expression of 12 and 27 genes were identified in the Co-NEC and Si-NEC group, i.e., Co-DEGs and Si-DEGs, respectively (Fig. 3a, b; Additional file 1: Figure S3a, b; Additional file 3: Tables S2 and S3). Among the above NEC-related DEGs, only two DEGs were identified in both Si-NEC and Co-NEC groups, *HBB* and *TMEM167B*, encoding hemoglobin subunit beta and transmembrane protein 167 B respectively, both of which were downregulated in NEC-positive pigs. The subsequent qPCR analysis confirmed the downregulation of the *HBB* gene in Si-NEC groups (*p* < 0.05, Fig. 3d).

GO analysis of all these NEC-related genes revealed four biological processes in the hippocampus that might be related to NEC, including “leukocyte migration involved in inflammation response,” “regulation of nitric oxide biosynthetic process,” “regulation of tissue remodeling,” and “regulation of pH” (Fig. 3c; Additional file 3: Table S5). Most of the involved genes in these functions were upregulated only in Si-NEC pigs, except *HBB* (involved in the regulation of the nitric oxide biosynthetic process), which was downregulated in both NEC groups (Fig. 3c). Moreover, we identified 76 DEGs between Si-NEC versus Co-NEC group, and 23 and 3 of them were previously identified as the Si-NEC and Co-NEC-related genes, respectively (Fig. 3a, Additional file 3: Table S4 and Additional file 1: Figure S3c).

Several genes specific to Si-NEC pigs from each category of biological processes were then validated by qPCR. Thus, upregulation of *PDK4* and *S100A8* in Si-NEC pigs was confirmed (*p* < 0.05, Fig. 3d). The gene mostly affected, *S100A8*, showed a sevenfold upregulation by RNA-seq and a threefold upregulation by qPCR in Si-NEC pigs (*p* < 0.05, Fig. 3d, Additional file 3: Table S3). In addition to genes within the enriched biological processes, the changes in expression of several other disease-specific Si-NEC and Co-NEC DEGs identified by RNA-seq were further verified by qPCR, including upregulation of *IER3* and *TXNIP* (*p* < 0.01 for both genes) and downregulation of *OPALIN* in Si-NEC pigs (*p* < 0.05) and upregulation of *TF* in Co-NEC pigs (*p* = 0.005, Fig. 3d). Interestingly, *ALAS2* mRNA, encoding an enzyme involved in the heme biosynthesis pathway, was downregulated in Si-NEC pigs (*p* = 0.02, Fig. 3d), thus correlating with the downregulation of *HBB* expression.

### Altered expression of inflammatory-, hypoxia-, and plasticity-related genes in the hippocampus of Si-NEC pigs

**Fig. 4:**
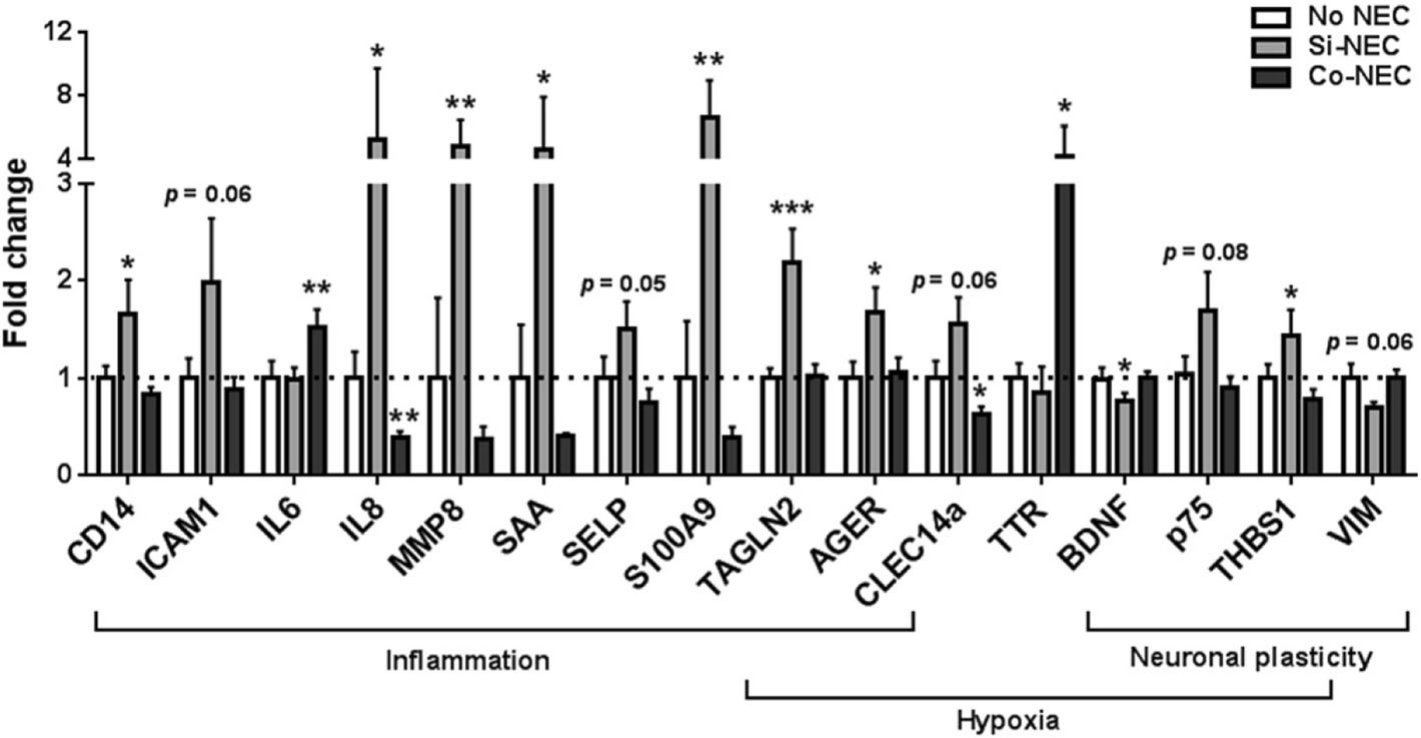
Inflammation- and hypoxia-related hippocampal gene expression in pigs with or without NEC measured by qPCR. Values are means ± SEMs, *n* = 9–16 per group. *p < 0.05; **p < 0.01; ***p < 0.001 vs No NEC pigs (dashed line)

Since a number of affected pathways and validated genes were related to inflammatory responses (Fig. 3c, d; Additional file 3: Table S5), we further investigated the expression of inflammatory genes, i.e., *ICAM1*, *IL6*, *IL8*, *SAA*, *S100A9* [48], *P Selectin*, *AGER* encoding RAGE, *TAGLN2* encoding Transgelin 2 [49], *CD14* [50], and *TLR4*, in the hippocampus. The results showed that *AGER*, *CD14*, *IL8*, *MMP8*, *SAA*, *S100A9*, and *TAGLN2* were upregulated in the Si-NEC group (*p* < 0.05, Fig. 4), whereas *P Selectin* and *ICAM1* showed only a tendency to be upregulated in this group (*p* = 0.05 and *p* = 0.06, respectively). Inflammation is characterized by changes in tissue oxygen supply and generation of reactive oxygen and nitrogen species. Among the above upregulated pro-inflammatory genes in Si-NEC pig hippocampi, many have been shown to be associated with hypoxia [51–54], including *EDN1*, *PDK4*, *IER3*, *TXNIP* (Fig. 3d) and *AGER*, *CLEC14a*, *TAGLN2*, *TTR*, *p75*, and *THBS1* (Fig. 4). Importantly, among all aforementioned genes only *IL6* and *TTR* were upregulated in the Co-NEC group (Fig. 4), suggesting that the hippocampal response to NEC differed between Si-NEC and Co-NEC pigs. Of note, a number of qPCR-validated genes related to inflammation and hypoxia, including *IL-6*, *p75*, *THBS1*, and *VIM* have been previously shown to regulate physiological neuronal plasticity, learning, and memory [54–56]. Collectively, our results show that NEC, especially in the small intestine, is associated with immediate changes in inflammatory- and hypoxia-related gene expression in the developing hippocampus.

### NEC is associated with microglia activation

**Fig. 5:**
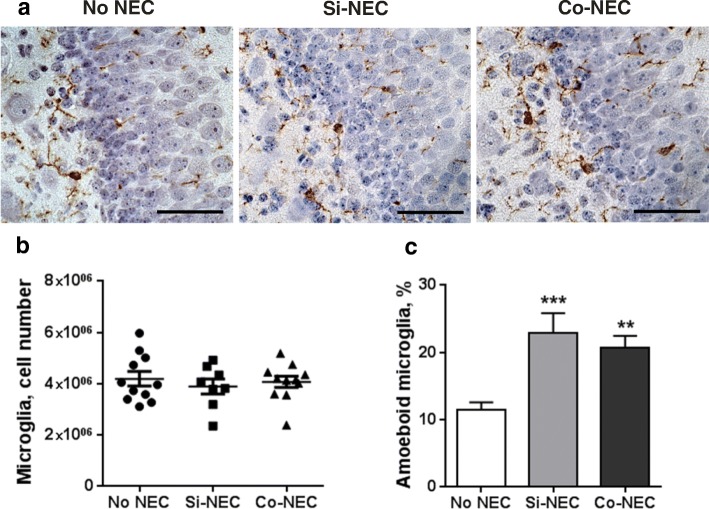
NEC lesions were associated with microglial activation in the hippocampus. **a** Representative images showing the Iba1-positive microglial cells in the hippocampus of No NEC, Si-, and Co-NEC pigs. Scale bar is 50 μm. **b** Scatter plot showing stereological quantification of total number of microglia with horizontal bars representing means ± SEMs. **c** Ratio of amoeboid cells to the total number of Iba1-positive cells. Values are means ± SEMs. **p < 0.01; ***p < 0.001 vs No NEC pigs

We further proceeded to study whether changes in the CSF cytokine profile and hippocampal gene expression in NEC pigs were related to the activation of resident microglia. The hippocampi from pigs with or without NEC were stained for microglia marker Iba1, and the number and morphology of microglial cells was analyzed using an unbiased stereological approach (Fig. 5a). There were no statistical differences in total microglia number among groups (*p* > 0.05, Fig. 5b). Analysis of the morphological phenotype of Iba1-positive cells showed that microglia with ramified processes were the most prominent type in all three groups. However, microglia with the amoeboid phenotype [38] were also observed, and their number increased significantly in hippocampi of both NEC groups (*p* < 0.01; Fig. 5c). Thus, despite there was no change in the total number of Iba1-positive cells in Si-NEC and Co-NEC, both NEC groups showed marked increases in the proportion of pro-inflammatory amoeboid microglia. Neither the number of the Iba + microglial cells nor the proportion of amoeboid cells in the hippocampus were affected by sex (*p* = 0.24, *p* = 0.31, respectively).

### S100A9 and the S100A8/S100A9 heterocomplex promote neurite outgrowth

**Fig. 6:**
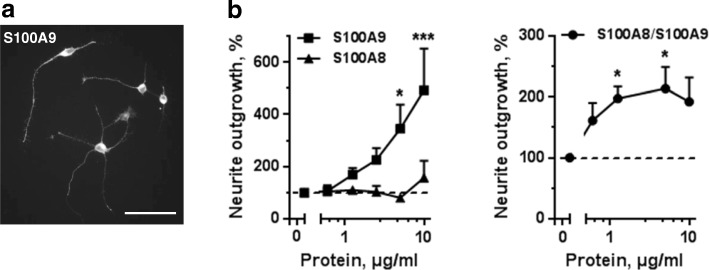
Neurotrophic effects of serially diluted S100A8, S100A9, and their heterocomplex of S100A8/S100A9 in primary hippocampal neurons. **a** Representative image illustrating S100A9-induced neurite outgrowth. **b** Quantification of neurite outgrowth from neurons stimulated with S100A9, S100A8, and S100A8/S100A9 hetero-complex for 24 h. Data were produced from four to six independent experiments and normalized to unstimulated controls which were set to 100% (dashed line). Values are means ± SEMs. *p < 0.05; ***p < 0.001 versus unstimulated controls

Finally, we further investigated the potential biological effects of proteins encoded by highly NEC-associated hippocampal genes, namely S100A8 and S100A9 (calgranulins). Several other members of the S100 family, including S100A4 and S100A12, have previously been shown to promote neurite outgrowth from different types of primary neurons [57, 58]. Consequently, we investigated whether S100A8, S100A9, and the S100A8/S100A9 heterocomplex (calprotectin) affected developing hippocampal neurons in vitro. Primary neurons were stimulated with serially diluted S100A9, S100A8, and S100A8/A9 for 24 h. As shown in Fig. 6a, b, S100A9 and S100A8/A9 promoted neurite outgrowth in a dose-dependent manner, while no effect of S100A8 on neurite extension could be detected. The data further support that inflammation-induced proteins may affect developing hippocampal neurons.

## Discussion

In contrast to term infants, a preterm infant has an immature gastrointestinal tract, brain, and immune system and such infants have high risks of both NEC and brain damage in early life. Severe NEC is known to be strongly associated with abnormalities in white and gray matters and poor neurodevelopmental outcomes which develop later in life [7, 59, 60]. For obvious reasons, the brain samples from neonates are inaccessible; thus, it remains unclear whether NEC lesions are associated with immediate adverse responses in the developing brain. Understanding the early NEC-related events in the brain is important to identify new protective strategies for preterm infants during the immediate neonatal period. Taking advantage of a clinically-relevant preterm pig NEC model, we now document that acute NEC located in the small intestine is associated with decreased physical activity and changes to hippocampal gene expression. NEC lesions that include the small intestine (with or without colon lesions) were associated with more profound responses in the developing hippocampus, relative to NEC lesions present exclusively in the colon region. This observation is consistent with the experience from human infants in which NEC diagnosis requiring subsequent tissue resection usually involves the presence of severe NEC lesions in the small intestine [27].

Exposure of rodent hippocampal neurons to CSF samples from NEC pigs promoted neuronal differentiation in vitro. The differentiation of neuronal precursor cells towards polarized neurons occurs via well-defined morphological steps and includes neurite outgrowth followed by neurite differentiation into dendrites and axons, and synapse formation. This differentiation is orchestrated by a number of factors, including immune-related molecules [61, 62], whose basal expression is much higher in the developing brain, compared with the mature brain [14, 63, 64]. In our study, the observed increase in neurite outgrowth and branching of neurites induced by CSF from NEC-affected animals may therefore be a consequence of elevated levels of pro-inflammatory cytokines (e.g.,VEGF [45], MMP-8 [43], CINC-3 [65] (called CXCL2 in humans) and INF-γ [42]). The promotion of neurite outgrowth from hippocampal neurons was confirmed specifically for VEGF and CINC-3, which further supports the hypothesis that at least some inflammation-induced factors may affect neuronal plasticity. Consistent with this, two hippocampal inflammation-related genes, coding for S100A8 and S100A9, were upregulated in Si-NEC pigs. These proteins form homodimers (Calgranulins) and a heterocomplex (Calprotectin) and are shown to interact and regulate the biological activities of receptors for advanced glycation end products (RAGE) and TLR4 (gram-negative bacterial liposaccharide receptor). Although S100A8 and S100A9 were upregulated within the CNS in response to different pathological conditions [66, 67], their role in brain development is poorly understood. We now first show that the extra-cellular application of S100A9 or S100A8/A9 (but not S100A8 alone) dose-dependently promotes neurite outgrowth from primary hippocampal neurons, further pointing to the stimulatory effect of inflammatory factors on neuronal plasticity. In addition to S100A8/A9, the neuritogenic and neuroprotective effects may also be induced by *ADAM8* [68] which was upregulated specifically in the Si-NEC group. Likewise, hippocampal upregulation of transferrin (*TF*), particularly in the Co-NEC group, confirms previous results on neurite outgrowth in vitro [69]. Apart from affecting neural maturation, pro-inflammatory cytokines are also well known to interfere with oligodendrocyte maturation and myelination processes [47]. In line with this notion, Si-NEC preterm pigs showed downregulation of *OPALIN*, a marker of mature oligodendrocytes [70]. Based on our results, we suggest that inflammation-induced factors may have effects on immature neurons to enhance their maturation and confer neuronal plasticity which might lead to redirection of the neural network formation and consequently to neurodevelopmental disorders. This is consistent with the notion that neonatal inflammation is closely associated with neurological disorders such as schizophrenia, autism spectrum disorder, and Rett syndrome [64, 71]. We speculate that peripheral inflammation in the gut may lead to dysregulation of neuronal differentiation and formation of synaptic networks and have long-term neurodevelopmental consequences.

It is widely accepted that inflammation and oxidative stress responses often occur simultaneously, but it is unclear if hypoxia-activated genes have consecutive, correlative, or causal roles. The Si-NEC DEGs were highly enriched with upregulated genes related to hypoxia (see the “Results” section) and downregulated transcripts encoding *HBB* and its synthetase *ALAS2*. Hemoglobin beta (HBB) is known to be expressed in neurons [72], hippocampal astrocytes, and mature oligodendrocytes [73] and is neuroprotective against oxidative and nitrosative stresses [74]. HBB may also support neuronal metabolism via epigenetic control of histones [75] and neuronal mitochondria functions [72]. Thus, Si-NEC-related downregulation of *HBB* may therefore uncover neurons for potential oxidative and NO stress and inhibit hippocampal mitochondrial functions. Together, inflammation- and hypoxia-related events appear responsible for effects of Si-NEC on the developing hippocampus.

We observed an increase in the amoeboid microglia population in the hippocampus of NEC pigs, which constituted about 10–20% of the total microglial cells. However, we did not observe difference between groups in the total number of microglia. The relative low abundance of activated microglia may be explained by the upregulation of *ADM*, as adrenomedullin downregulates LPS-induced microglia activation and decreases the production of pro-inflammatory cytokines in vitro [76]. Further, upregulated expression of *S100A8* and *S100A9*, and of their RAGE receptor, may play a role, as soluble RAGE can function as a decoy receptor attenuating the pro-inflammatory effects of S100 proteins. Finally, NEC-related upregulation of *USP18* (Fig. 3a, Additional file 3: Table S3), encoding ubiquitin-specific protease 18, a negative regulator of microglia activation [77], therefore may counteract effect of NEC on microglia activation.

Overall, we observed more profound changes in hippocampal gene expression in Si-NEC pigs than in Co-NEC pigs. These changes suggest potential neuroinflammation, hypoxia, and oxidative distress, which are more evident in the pigs with NEC lesions in the small intestine than in the colon. This may be because the small intestine is more vulnerable to insults in early life than the colon [78, 79], and small intestinal NEC is more often linked to increased disease severity and mortality, less nutrient absorption, and high risk of bacterial translocation [28]. Some of the pigs in Si-NEC group also had NEC lesions in the colon, yet the hippocampal gene expression profile of these pigs were not different from the pigs only having NEC in the small intestine (Additional file 1: Figure S4). Apart from neonatal inflammation, NEC-associated malnutrition and poor growth are also risk factors for brain injuries and neurodevelopmental impairments in preterm infants [80–82]. However, nutritional factors may not play a critical role in the present study considering the continuous parenteral nutrition supply for all animals and body weight was not different among the groups.

## Conclusion

Our results help explain why NEC in early life is linked to poor neurological outcome later in preterm infants. We show that especially NEC lesions in the small intestine are associated with altered physical activity and with profound effects on hippocampal gene expression, related to inflammation and hypoxia. CSF from NEC-positive pigs promotes neurite outgrowth and neuronal branching, thus suggesting that peripheral inflammation may interfere with neuronal maturation. The results show the importance of providing brain-protective interventions to hospitalized preterm infants that experience severe NEC lesions. Further research is required to investigate whether NEC-associated inflammation in the developing brain adversely affects neural network formation and cognitive function more long term.

## Additional files

Additional file 1: **Figure S1.** Representative images of the small intestine and the colon with or without NEC lesions. The depicted lesions in the small intestine corresponds to NEC score 6, and lesions in the colon correspond to NEC score 4. **Figure S2.** Pro-inflammatory protein profiles in CSF from Si-NEC, Co-NEC, and No NEC pigs. Four to seven CSF samples from each group were equally pooled and 100 μl of undiluted CSF mixture from each group was applied to the pre-configurated sandwich Rat Cytokine Array G2 (AAR-CYT-G2–8, RayBiotech, USA according to the manufacturer’s protocol. The chip was scanned with a laser scanner using the Cy3 channel with background subtraction and data normalization among sub-arrays. In the array, antibodies were spotted twice, providing two technical replicates for each of the 34 tested proteins, using a standard chip layout (www.raybiotech.com/g-series-rat-cytokine-array-g2-4.html). Values are the mean signal intensity normalized to No NEC. **Figure S3.** Bar-graphs summarizing the RNA-seq results for differentially expressed hippocampal genes between the (**a**) Co-NEC vs No NEC, (**b**) Si-NEC vs No NEC, and (**c**) Si-NEC vs Co-NEC groups (log_2_ fold changes). **Figure S4.** Hippocampal gene expression profile is similar between pigs diagnosed with small intestinal NEC and pigs with both small intestinal and colonic NEC, which are both different from Co-NEC group. PCA based on relative gene expression measured by microfluidic qPCR analysis. (PPT 7404 kb)

Additional file 2: **Table S1.** Characteristics of preterm pigs included in the study. (DOCX 14 kb)

Additional file 3: **Table S2.** Differentially expressed genes (DEGs) between Co-NEC and No-NEC groups. Listed are all the identified DEGs (*q* value < 0.2), with corresponding location, average FPKM in each group, the (base 2) log of the fold change, the value of the test statistic from Cufflinks, the uncorrected *p*-value and FDR-adjusted p-value (*q* value). **Table S3.** Differentially expressed genes (DEGs) between Si-NEC and No-NEC groups. Listed are all the identified DEGs (*q* value < 0.2), with corresponding location, average FPKM in each group, the (base 2) log of the fold change, the value of the test statistic from Cufflinks, the uncorrected p-value and FDR-adjusted p-value (*q* value). **Table S4.** Differentially expressed genes (DEGs) between Si-NEC and Co-NEC groups. Listed are all the identified DEGs (*q* value < 0.2), with corresponding location, average FPKM in each group, the (base 2) log of the fold change, the value of the test statistic from Cufflinks, the uncorrected p-value and FDR-adjusted p-value (*q* value). **Table S5.** Gene ontology enrichment analysis of DEGs between NEC and No-NEC groups. Listed are enriched gene ontology terms, corrected *p*-values and involved genes. **Table S6.** List of gene names, primer sequences and amplicon length of all primers, used in the study. *, reference genes. (XLSX 61 kb)

## Abbreviations

CINC: Cytokine-induced neutrophil chemoattractant
CRP: C-reactive protein
CSF: Cerebrospinal fluid
DEG: Differentially expressed genes
ELISA: Enzyme-linked immunosorbent assay
GAP-43: Growth-associated protein GAP-43
HBB: Hemoglobin beta
ICAM-1: Inter-cellular adhesion molecule 1
MIP: Macrophage inflammatory protein
NEC: Necrotizing enterocolitis
PBS: Phosphate-buffered saline
PCR: Polymerase chain reaction
RAGE: Receptor for advanced glycation end products
RIN: RNA integrity number
SEM: Standard error of the mean
TIMP: Tissue inhibitor of metalloproteinase
VEGF: Vascular endothelial growth factor
