# Supplementary material for: Necrotizing enterocolitis is associated with acute brain responses in preterm pigs
Source: J Neuroinflammation. 2018 Jun 9;15:180. doi: 10.1186/s12974-018-1201-x (PMC5994241; doi:10.1186/s12974-018-1201-x)
Supplement: Supplementary file 1 — Figure S1. Representative images of the small intestine and the colon with or without NEC lesions. The depicted lesions in the small intestine corresponds to NEC score 6, and lesions in the colon correspond to NEC score 4. Figure S2. Pro-inflammatory protein profiles in CSF from Si-NEC, Co-NEC, and No NEC pigs. Four to seven CSF samples from each group were equally pooled and 100 μl of undiluted CSF mixture from each group was applied to the pre-configurated sandwich Rat Cytokine Array G2 (AAR-CYT-G2–8, RayBiotech, USA according to the manufacturer’s protocol. The chip was scanned with a laser scanner using the Cy3 channel with background subtraction and data normalization among sub-arrays. In the array, antibodies were spotted twice, providing two technical replicates for each of the 34 tested proteins, using a standard chip layout (www.raybiotech.com/g-series-rat-cytokine-array-g2-4.html). Values are the mean signal intensity normalized to No NEC. Figure S3. Bar-graphs summarizing the RNA-seq results for differentially expressed hippocampal genes between the (a) Co-NEC vs No NEC, (b) Si-NEC vs No NEC, and (c) Si-NEC vs Co-NEC groups (log2 fold changes). Figure S4. Hippocampal gene expression profile is similar between pigs diagnosed with small intestinal NEC and pigs with both small intestinal and colonic NEC, which are both different from Co-NEC group. PCA based on relative gene expression measured by microfluidic qPCR analysis. (PPT 7404 kb) [file 12974_2018_1201_MOESM1_ESM.ppt]

## Slide 1
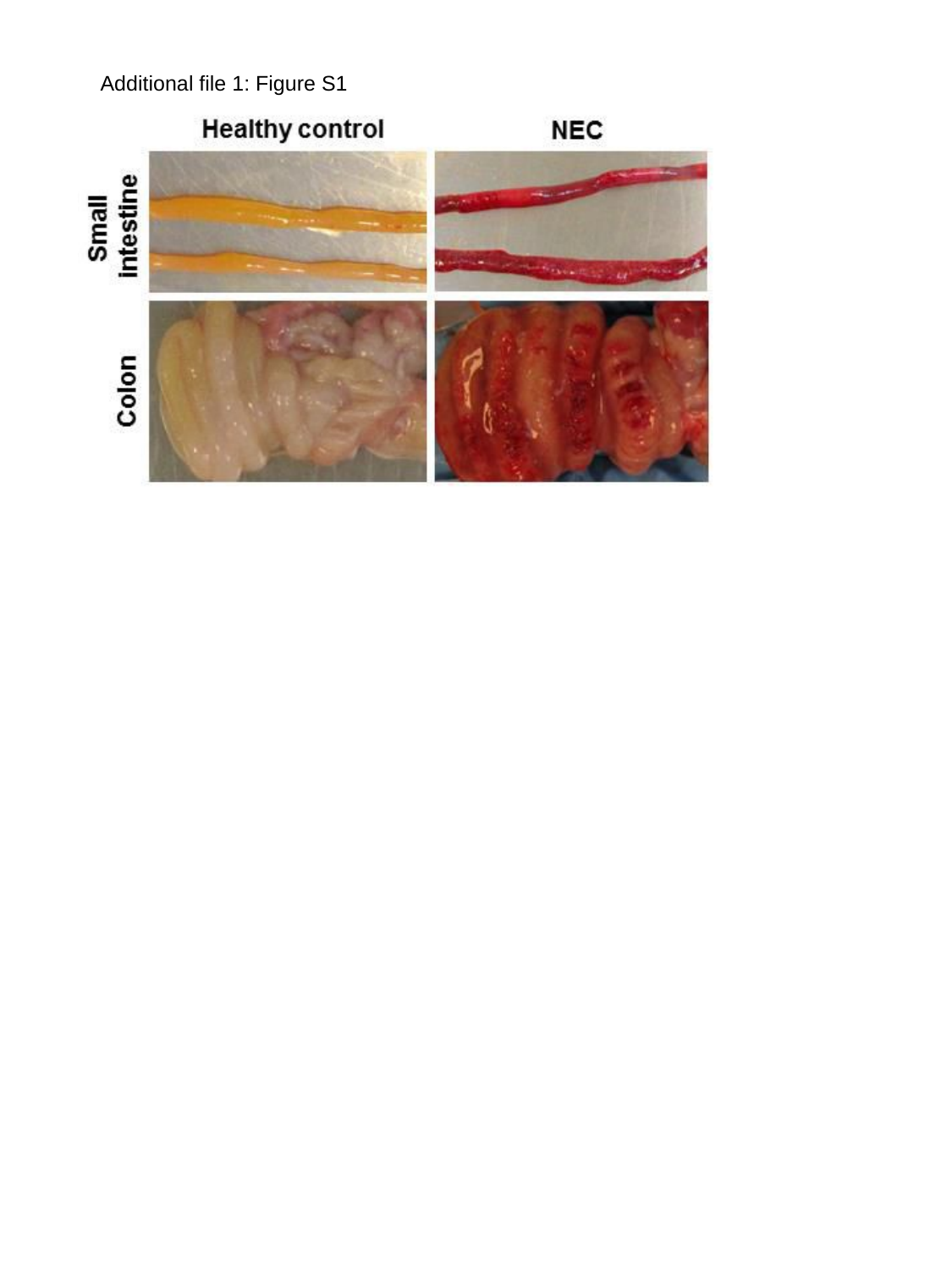

Additional file 1: Figure S1

## Slide 2
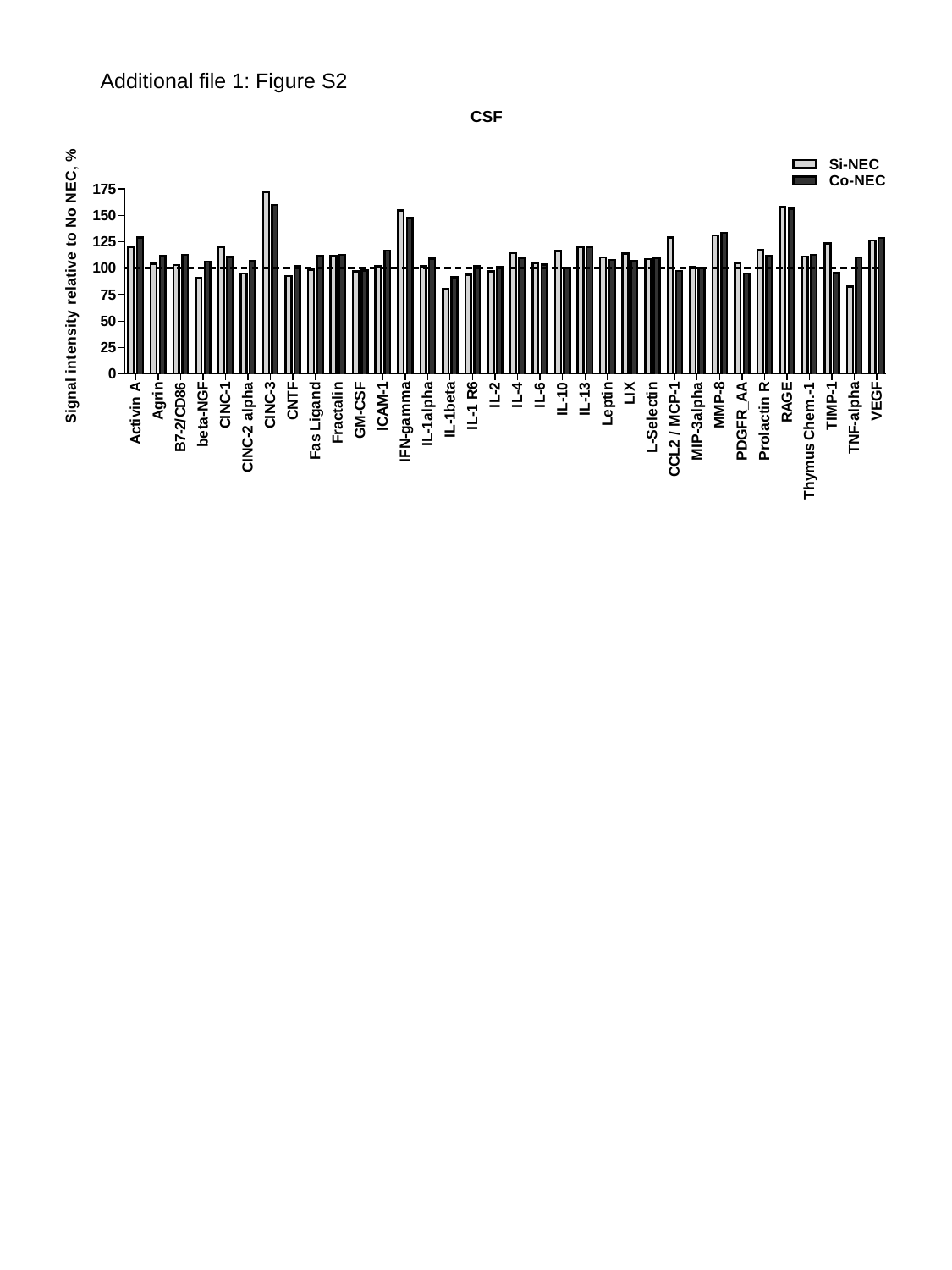

Additional file 1: Figure S2
CSF

## Slide 3
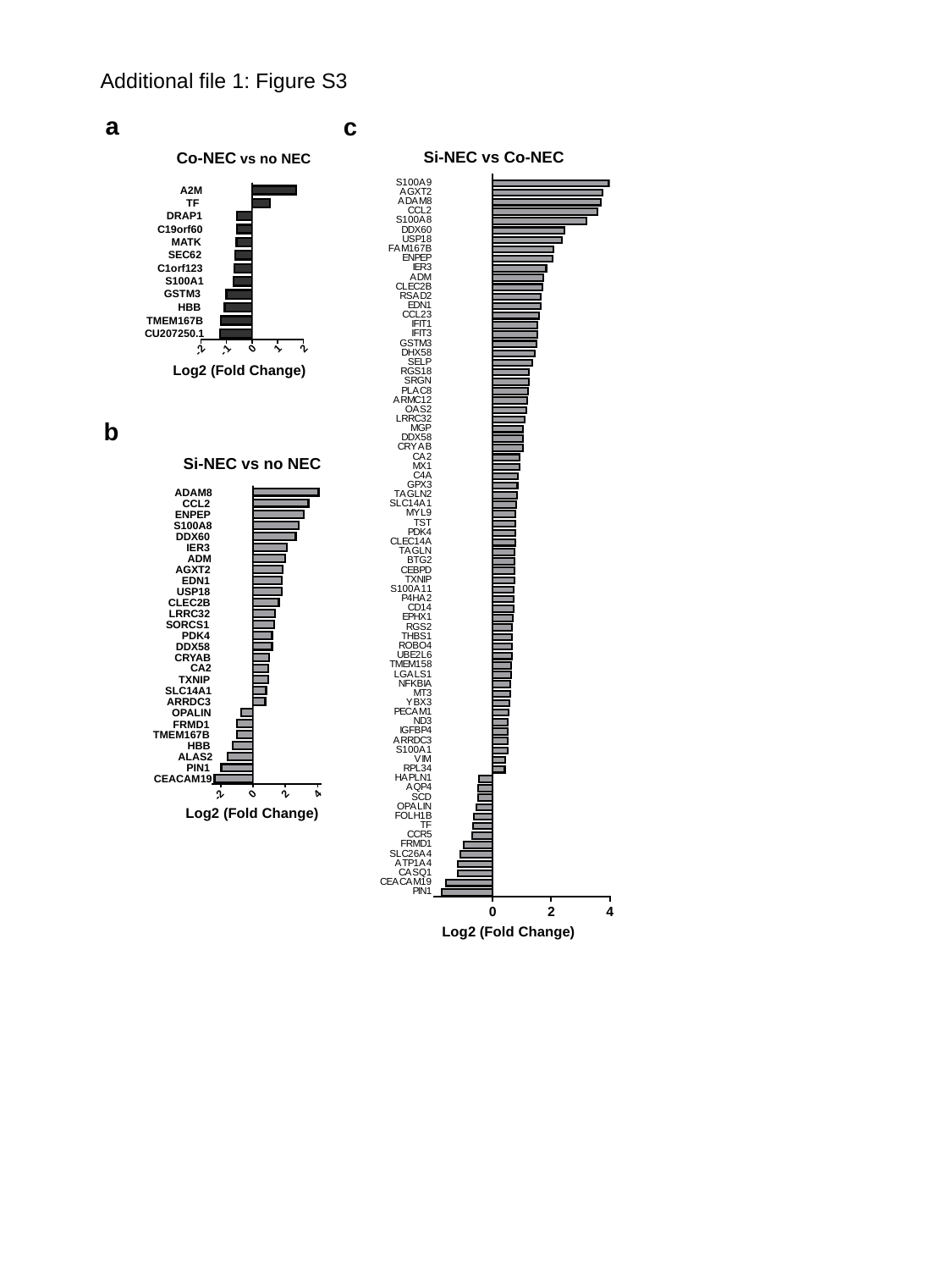

Additional file 1: Figure S3
a
c
Si-NEC vs Co-NEC
Log2 (Fold Change)
Co-NEC vs no NEC
A2M
TF
DRAP1
C19orf60
MATK
SEC62
C1orf123
S100A1
GSTM3
HBB
TMEM167B
CU207250.1
2
1
0
1
2
-
-
Log2 (Fold Change)
b
Si-NEC vs no NEC
ADAM8
CCL2
ENPEP
S100A8
DDX60
IER3
ADM
AGXT2
EDN1
USP18
CLEC2B
LRRC32
SORCS1
PDK4
DDX58
CRYAB
CA2
TXNIP
SLC14A1
ARRDC3
OPALIN
FRMD1
TMEM167B
HBB
ALAS2
PIN1
CEACAM19
2
0
2
4
-
Log2 (Fold Change)

## Slide 4
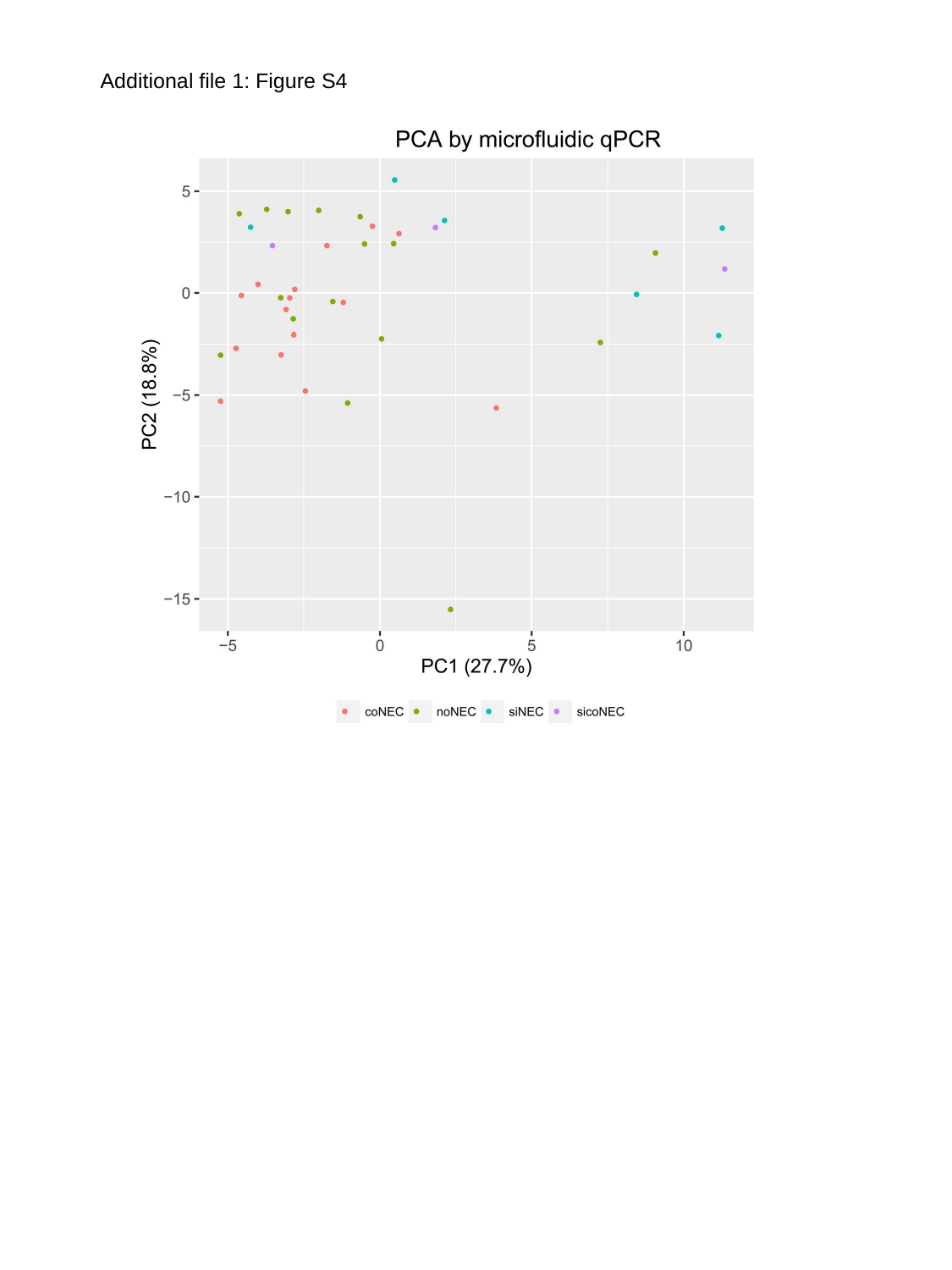

Additional file 1: Figure S4
