# Supplementary material for: Necrotizing enterocolitis is associated with acute brain responses in preterm pigs
Source: J Neuroinflammation. 2018 Jun 9;15:180. doi: 10.1186/s12974-018-1201-x (PMC5994241; doi:10.1186/s12974-018-1201-x)
Supplement: Supplementary file 2 — Table S1. Characteristics of preterm pigs included in the study. (DOCX 14 kb) [file 12974_2018_1201_MOESM2_ESM.docx]

**Table S1. Characteristics of preterm pigs included in the study.**

|  | **No NEC** | **Si NEC** | **Co NEC** |
| --- | --- | --- | --- |
| Birth weight, g | 904 ± 31 | 908 ± 73 | 929 ± 37 |
| Final body weight, g | 906 ± 34 | 1061 ± 94 | 1034 ± 44 |
| Female:Male | 20:31 | 7:6 | 20:14 |
| Number of pigs | 51 | 13 (5)^1^ | 34 |
| Time taken for first eye opening, h | 25 ± 3 | 45 ± 12 | 27 ± 3 |
| Time taken for first stand, h | 46 ± 6 | 48 ±11 | 42 ±5 |
| Time taken for first walk, h | 51 ± 6 | 54 ± 10 | 57 ± 8 |
| Plasma glucose, mM | 5.2 ± 0.6 | 5.9 ± 0.6 | 9.2 ± 2.3 |

^1^The number in the bracket represents number of pigs with NEC score≥4 in both the small intestine and the colon regions.
